# Tumor-immune Co-evolution in hepatocellular carcinoma and its implication for immunotherapy resistance

**DOI:** 10.1016/j.iliver.2022.02.003

**Published:** 2022-03-09

**Authors:** Zhiyuan Fang, Huayu Yang, Yilei Mao

**Affiliations:** Department of Liver Surgery, Peking Union Medical College (PUMC) Hospital, PUMC & Chinese Academy of Medical Sciences (CAMS), Beijing, 100730, China

## 1 Introduction

In recent years, investigators have witnessed great progress in understanding the tumor microenvironment (TME) and evolution, which has been accompanied by a substantial number of newly developed targeted therapies and immunotherapies. Tumors are capable of escaping immune surveillance in the context of both spontaneous immune response and immunotherapies, and these two immune evasion processes share many overlapping mechanisms, such as human leukocyte antigen loss of heterozygosity (HLA-LOH) and immunoediting [1]. For hepatocellular carcinoma (HCC), numerous treatment strategies targeting the TME have been proposed in recent years [2,3]. Among these strategies, immune-checkpoint inhibitors (ICIs) have been found to be a promising therapeutic choice [[4], [5], [6]]. The combination of atezolizumab (anti-programmed death-ligand 1, PD-L1) and bevacizumab (anti-vascular endothelial growth factor, VEGF) antibodies demonstrated significantly better overall and progression-free survival than the kinase inhibitor sorafenib in patients with unresectable HCC in a phase III trial [4], and this combination was established as a first-line treatment for advanced HCC. However, the response rate for ICIs was limited [[4], [5], [6]], and it was reported that nonalcoholic steatohepatitis-related HCC was less responsive to ICIs than other HCCs [7]. Given the clinical importance of tolerance to immunotherapy in HCC, tumor-immune co-evolution deserves more attention and may provide novel ideas for developing new combination therapies that target multiple mechanisms of immunotherapy resistance.

## 2 Interactions between tumor evolution and the immune microenvironment

Tumor evolution and the TME have been studied extensively in HCC. Genomic and epigenomic intratumor heterogeneity (ITH) is regarded as a hallmark of tumor evolution and has been linked to progression, prognosis, and recurrence of HCC [8,9]. However, knowledge of tumor evolution is difficult to directly translate into clinical treatment strategies because of the lack of targetable oncogene addiction and the potential toxicity when combining different drugs [10]. The landscape of the TME may be used as an HCC classification indicator with prognostic impact [11]. With the help of novel technologies, such as single-cell sequencing, infiltrating T cells in HCC were classified into 11 subsets and characterized by the enrichment of Tregs and exhausted CD8^+^ T cells [12]. A more comprehensive and dynamic landscape of immune cells in the TME and other associated tissues provide a deeper understanding of the roles and interactions of the TME components [13]. As the knowledge of both tumor evolution and the TME continues to grow, the frontiers of these two fields are beginning to intersect.

By combining the ideas of tumor evolution and the TME, one study demonstrated correlations between tumor cell heterogeneity and local immune status in HCC using multi-omics analysis [14]. The researchers found that the local immune status was less heterogeneous than the tumor cells, and phosphatidylinositol-3-kinase-Akt signaling and ketone body metabolism in tumor cells was associated with CD8^+^ T cell infiltration in the HCC microenvironment. Then, they conducted hierarchical clustering of immune cells and classified HCC into 3 immunophenotypic subtypes; cytokine and chemokine expression and the tumor cell metabolome also exhibited characteristics in parallel with the immunophenotypes. These results suggested the potential mutual impact of tumor and immune cells.

In another study, tumor transcriptomic diversity was measured in HCC and intrahepatic cholangiocarcinoma samples using single-cell transcriptomic analysis, and the degree of diversity was compared with the outcome of patients treated with ICIs [15]. Both the overall and progression-free survival of patients with higher tumor transcriptomic diversity were significantly poorer than patients with lower diversity, indicating the impact of ITH on immunotherapy responsiveness. These data also established a correlation between higher levels of VEGF expression in more diverse tumors and TME polarization, including lower T cell cytolytic activity, which was consistent with their hypothesis that tumor-derived VEGF drives microenvironmental reprogramming. Taken together, the relationship between ITH and immune exhaustion justifies the speculation that immunosuppression allows more mutations and favors tumor diversification.

The interactions between tumor and immune cells that contributed to HCC immune evasion were extensively studied using immunogenomics. The regions predicted to have less immunogenic neoepitopes were found to have a greater tumor infiltrating lymphocyte (TIL) burden, suggesting that the adaptive immune response mediated the editing of tumors to be much less immunogenic [16]. Another study examined immunogenomic features of multifocal HCC from either intrahepatic metastasis or multicentric occurrence origin [17]. Immunoediting existed only in some multicentric occurrence cases with high TIL infiltration. The immune evasion modes for intrahepatic metastasis tumors included PD-1^+^ M2 macrophages that impeded T cell infiltration and HLA-LOH that interfered with neoantigen presentation under high TIL pressure. The different immune tolerance patterns presented a corresponding relationship between the tumor cell and TME co-evolution. Given the ITH within the TME, immune-ITH has been proposed as an indicator of tumor evolution and can be used to predict disease prognosis [18]. The immune-ITH was positively correlated with the extent of immunosuppression and poor prognosis. With regard to tumor evolution, tumors with higher immune-ITH had greater immune cell exhaustion in the TME, which provided the tumor cells with greater freedom to accumulate mutations. Tumors with lower immune-ITH experienced higher negative selection pressure and evaded immune surveillance through other mechanisms, such as immunoediting and/or HLA-LOH.

## 3 TME evolution during immunotherapy

The application of immunotherapy provides an opportunity to observe the adaptive evolution of tumors in response to drastic changes in the immune microenvironment within a relatively short period. The single-cell landscape was determined in liver tumor samples from patients enrolled in various clinical trials of ICIs [19]. ITH correlated with prognosis and TME polarization as described in previous studies. Furthermore, the secreted phosphoprotein 1 (*SPP1*) gene was identified as a potential driver of tumor evolution. Much stronger interactions between malignant cells and T cells were observed in tumors with greater diversity, and SPP1-CD44 was described as a principal interaction pair. The tumor and TME co-evolved in response to immunotherapy, and SPP1 may have acted as an important regulator in this co-evolution.

Because immunotherapy has been used against HCC for a relatively short period, studies on the mechanisms of tumor evolution during immunotherapy remain scarce. However, studies of the TME in HCC and investigations of other tumor types have provided insights into the immunotherapy resistance of HCC. Even in tumors with high PD-1/PD-L1 expression, anti-PD-1/PD-L1 treatment alone may not be effective because of low TIL numbers or tumor cell immunoediting and HLA-LOH. Furthermore, according to the co-evolution theory, in the TME activated by ICIs, tumor cells also undergo adaptive evolution, and tumor cell clones that have withstood the negative selection pressure may lead to rapid drug resistance and relapse once they become dominant. These theories may explain why current single-agent ICI therapies have not yielded satisfactory results for HCC [20].

## 4 Immunotherapy that targets an evolving TME

To improve the shortcomings of single-agent ICI therapies, there are two routes of combination therapy that include combining multiple ICIs and other targeted drugs [3] as shown in Table 1.

**Table 1:** Combination strategies used in clinical trials of advanced hepatocellular carcinoma.

Anti-PD-1/PD-L1 agent	Other agent	Phase	Reference
Durvalumab (anti-PD-L1)	Tremelimumab (anti-CTLA-4)	I/II	Kelley et al[21], 2021
Nivolumab (anti-PD-1)	Ipilimumab (anti-CTLA-4)	I/II	Yau et al[22], 2020
Atezolizumab (anti-PD-L1)	Bevacizumab (anti-VEGF)	III	Finn et al[4], 2020
Nivolumab (anti-PD-1)	Cabozantinib (TKI)	I/II	Yau et al[23], 2020
Pembrolizumab (anti-PD-1)	Lenvatinib (TKI)	Ib	Finn et al[24], 2020

CTLA-4, cytotoxic T-lymphocyte-associated protein 4; PD-1, programmed death-1; PD-L1, programmed death-ligand 1; TKI, tyrosine kinase inhibitor; VEGF, vascular endothelial growth factor.

A clinical trial of a single, high priming dose of tremelimumab (anti-cytotoxic T-lymphocyte-associated protein 4) combined with a continuous dose of durvalumab (anti-PD-L1) showed that the first dose of tremelimumab produced a proliferative burst of CD4^+^ and CD8^+^ T cells and, thus, may have created a better immune microenvironment for durvalumab to effectively function [21].

Pre-existing immunosuppressive components of the tumor-immune microenvironment are selected during immunotherapy. Therefore, it is reasonable to use a combination of targeted drugs against these components, such as VEGF, transforming growth factor-β, or indoleamine 2,3-dioxygenase, which are characteristic of HCC [3]. Combination immunotherapies with anti-VEGF antibody and tyrosine kinase inhibitors have achieved some encouraging results in clinical trials, which have included increased antigen presentation, increased activation and infiltration of CD8^+^ T cells, reduced expression of immunosuppressive components, and direct anti-tumor effects [25].

Other cases that do not respond well to current ICI therapies can be studied further from the perspective of tumor-immune co-evolution, such as nonalcoholic steatohepatitis-related HCC, which bears special resident-like activated CD8^+^ cells that cause hepatocyte damage, hepatic fibrosis, and decreased anti-tumor immunity [[7], [26], [27]]. More advanced combination immunotherapies are needed to rebuild an anti-tumor TME based on a more complicated native situation. Other strategies using T cells that directly target the evolved tumor cell clones, such as chimeric antigen receptor T cells, TILs, and neoantigen vaccines, may also promote the reconstitution of a TME favoring T cell infiltration and activation. For tumors with variable tumor-immune interactions, selection of personalized combination immunotherapy regimens may be possible in the future if the exact pathological components in the TME are demonstrated by immunophenotyping.

## 5 Conclusion

In conclusion, tumor-immune co-evolution in HCC tends to favor immune evasion and tumor progression in response to autogenous anti-tumor immunity and exogenous immunotherapy. Strong immunological stress promotes the evolution of tumor cells toward a less immunogenic phenotype, and tumor cells can also cause TME reprogramming which leads to reduced tumor cytotoxicity. As a result, patients may benefit more from combination immunotherapy if both the TME and tumor cell evolution are taken into consideration. Clinical trials of new combination immunotherapies and the development of novel clinically applicable immunotherapeutic approaches blocking tumor-immune co-evolution are desirable. Furthermore, many of the details and mechanisms of the interactions between tumor cells and the TME remain unknown, and the discovery of more druggable targets will be required.

## Declaration of competing interest

The author(s) declared no potential conflicts of interest with respect to the research, authorship, and/or publication of this article.
